# Fully Automatic Liver and Tumor Segmentation from CT Image Using an AIM-Unet

**DOI:** 10.3390/bioengineering10020215

**Published:** 2023-02-06

**Authors:** Fırat Özcan, Osman Nuri Uçan, Songül Karaçam, Duygu Tunçman

**Affiliations:** 1 Department of Mechatronics Engineering, Faculty of Technology, Kayalı Campus, Kırklareli University, 39100 Kırklareli, Turkey; 2 Faculty of Applied Sciences, Altınbaş University, Mahmutbey Dilmenler str., 26, 34217 Istanbul, Turkey; 3 Departman of Radiation Oncology, Cerrahpaşa Medical School, Cerrahpaşa Campus, İstanbul University-Cerrahpaşa, 34098 Istanbul, Turkey; 4 Radiotherapy Program, Vocational School of Health Services, Sultangazi Campus, İstanbul University-Cerrahpaşa, 34265 Istanbul, Turkey

**Keywords:** liver segmentation, tumor segmentation, U-Net, deep learning, computed tomography (CT), AIM-Unet

## Abstract

The segmentation of the liver is a difficult process due to the changes in shape, border, and density that occur in each section in computed tomography (CT) images. In this study, the Adding Inception Module-Unet (AIM-Unet) model, which is a hybridization of convolutional neural networks-based Unet and Inception models, is proposed for computer-assisted automatic segmentation of the liver and liver tumors from CT scans of the abdomen. Experimental studies were carried out on four different liver CT image datasets, one of which was prepared for this study and three of which were open (CHAOS, LIST, and 3DIRCADb). The results obtained using the proposed method and the segmentation results marked by the specialist were compared with the Dice similarity coefficient (DSC), Jaccard similarity coefficient (JSC), and accuracy (ACC) measurement parameters. In this study, we obtained the best DSC, JSC, and ACC liver segmentation performance metrics on the CHAOS dataset as 97.86%, 96.10%, and 99.75%, respectively, of the AIM-Unet model we propose, which is trained separately on three datasets (LiST, CHAOS, and our dataset) containing liver images. Additionally, 75.6% and 65.5% of the DSC tumor segmentation metrics were calculated on the proposed model LiST and 3DIRCADb datasets, respectively. In addition, the segmentation success results on the datasets with the AIM-Unet model were compared with the previous studies. With these results, it has been seen that the method proposed in this study can be used as an auxiliary tool in the decision-making processes of physicians for liver segmentation and detection of liver tumors. This study is useful for medical images, and the developed model can be easily developed for applications in different organs and other medical fields.

## 1. Introduction

Medical image segmentation, which has an important place in image analysis studies, is of great importance for doctors to make computer-aided diagnoses in the field of medicine [1]. Segmentation of the relevant organ or region from images obtained from different medical fields, especially in the field of radiology, is important in planning the early diagnosis and treatment of diseases. Compared with other clinics, the application of artificial intelligence (AI) in radiotherapy has been rapidly developed in recent years. Advanced treatment techniques such as intensity-modulated radiotherapy (IMRT) and volumetric modulated arc therapy (VMAT) have become standards in radiation oncology. These advanced techniques also required contouring of tumor volume and especially normal organs around the tumor (organs at risk-OARs). Clinicians delineate these structures on treatment planning systems using computers. The manual delineation of these volumes requires long-term accumulation experience for clinicians. This process causes inter- and intra-observer variability and is time-consuming. Deep-learning auto-segmentation could be a useful time-saving tool to optimize workload and resources in radiation therapy. The liver is one of the dose-limiting organs in radiotherapy for thoracic and abdominal tumors, so accurate contouring of the liver region is important. Ahn et al. demonstrated that a deep learning framework could be used more effectively and efficiently compared to atlas-based auto-segmentation for most OARs in human liver cancer [2].

Computed tomography (CT) is a frequently used imaging technique for the early detection of liver organ size, lesions, or tumors [3]. It consists of two related operations, even if any image is not segmented. The first is to find the relevant region, and the second is to define the boundaries of the region. Anomalies in the shape and texture of the liver and visible lesions on CT are important findings for disease progression in primary and secondary liver tumor disease. Manual or semi-manual techniques are routinely applied in clinics for detection and diagnosis [4]. However, these are subjective, operator-dependent, and very time-consuming. In addition, the correct determination of the relevant region varies depending on the experience and skill of the physician. Computer-aided methods have been developed to increase radiological productivity. Although computer-aided systems are indispensable aids in the determination of liver size and lesion size in order to reduce observer errors, recently, large shape differences among patients, abnormalities in tissues, low-density contrast between the liver and neighboring organs (pancreas, heart, stomach, etc.), and various pathologies (auto-segmentation of the liver and lesions is very difficult due to the presence of tumors, cirrhosis, cysts, etc.) [5]. In addition, pathological conditions such as fatty liver, iron deposition, fibrosis, and tumors may also expose the liver to anatomical deterioration in terms of tissue and signal intensity deterioration [6].

Despite these difficulties, extensive studies have been conducted to segment the liver and liver lesions in CT volumes. In this way, various interactive, automatic, and semi-automatic [7] methods have been developed. Three types of widely used image segmentation algorithms are generally employed for liver segmentation. The first of these are algorithms based on grayscale values [8,9,10], the second are algorithms based on statistical shape models [11,12,13], and the third are algorithms based on texture properties [14,15,16]. Since the development of deep learning methods, end-to-end liver segmentation has gained immense attention from various research groups. Deep learning-based CNNs (convolutional neural networks) can yield very good segmentation results, as deep learning techniques can easily learn high-level semantic features of the liver from images.

A series of studies based on liver segmentation and lesion detection machine learning were carried out from CT images. Most of these studies extracted low-level features [17,18] or mid-level features [19,20,21] from a preselected or predetermined ROI for the liver lesion classification task. Recently, studies have emerged that have achieved great success in a variety of challenging tasks, including the field of high-level feature representation in deep convolutional neural networks (DCNN) and liver lesion classification. There are many algorithms developed by researchers around the world for their work. Algorithms can be divided into several classes: 3D-based deep learning [22,23,24], 2D-based deep learning [25,26,27], statistical-based [27,28], and computer vision-based [29]. Among the developed models, U-Net, CE-net, Inception-v2, and versions are popular for two-dimensional segmentation, while U-Net and V-net are more preferred for 3D segmentation. Although deep learning provides better end-to-end liver segmentation, some new challenges have emerged that restrict the use of deep learning in clinics. Because liver slices generate volumetric data, it is difficult to use 3D spatial information of liver slices when using 2D CNN for liver segmentation.

AIM-Unet is a method that produces more successful feature maps about the image by using filters of different sizes on the image. Accordingly, it is seen that the AIM-Unet model can be used for the detection and segmentation of liver and tumors on CT images. In this study, an automated computer-assisted AIM-Unet model using the bases of the Unet and Inception models is proposed for high-accuracy detection and segmentation of liver and tumor on abdominal CT images in four datasets.

The main contributions of this study are:

1. Proposed a new hybrid Aim-Unet deep neural network, which is a combination of Unet and Inception v3 architectures, for liver and tumor segmentation.

2. Presented a fully automated and robust segmentation system that can segment liver and tumors.

3. An original dataset was created for liver segmentation with images taken from the hospital.

4. Three different datasets for the liver and two different datasets for the tumor were used, with various preprocessing, optimization algorithms, and loss functions, in order for the system to successfully segment both the liver and tumor areas.

## 2. Literature Review

The first neural network proposed for liver segmentation relied on pre- and post-processing to extract irrelevant regions and then extracted smooth borders with the help of morphological operations. However, the new Convolutional Neural Networks (CNNs) are data-driven algorithms that can be optimized end-to-end without hand-made feature engineering. For the first time in the scientific world, deep learning made a big impact in 2012. It won the Large-Scale Visual Recognition Competition, the largest competition in object identification, with convolutional neural network (CNN) that year. It is considered the fundamental architecture in deep learning. This is an incredible rise in deep learning. Convolutional neural networks (CNNs) have had great success with many object recognition problems in the computer vision community. This trend has been followed by many researchers, so it has been suggested that CNNs learn feature representations in the practice of liver and lesion segmentation. Tumor sites in the liver can occur in various sizes, shapes, textures, contrasts, and numbers. The frequently used deep learning model for segmenting medical images is U-Net. Researchers aimed to increase the performance of U-Net by making various changes and additions. Cornelio et al. [30] In a study they carried out in 2019, they modified the batch normalization that allows self-learning in each layer of the network. They also used different regularizers (Lasso regression and Ridge regression). In 2020, Thi et al. [31] developed a fully automatic tool to recognize the liver region from CT images based on a deep learning model, namely the Multiple Filter U-Net, MFUnet. According to Karthik et al. [32], a mechanism called attention gate was added to U-Net. Thanks to this mechanism, data from the expanding path and skip connection are first combined. After the combined data is stretched using the ReLu activation function, a linear transformation is applied. Finally, it is subjected to the sigmoid function. Irrelevant features are suppressed thanks to the attention coefficients used here. In 2022, Hille et al. [33] developed a hybrid network called SWTR-Unet, consisting of a pretrained ResNet, transformer blocks, and a common Unet-style decoder path. In the same year, Xiong et al. [34] proposed a weak tag-based Bayesian U-Net utilizing the Hough transform for optical disc segmentation. In Bayesian U-Net, the standard Gaussian is placed over the mesh weights. No changes have been made to the skip connection in this model.

Recently, AI applications such as convolutional neural network (CNN)-based automatic segmentation models have been shown to improve the consistency and efficiency of this process. These models typically classify each voxel in an image as belonging to OARs or target based on the characteristics of the location and density of the voxel and surrounding voxels. These AI models now outperform traditional auto-sculpting methods and reach the accuracy range of manual definitions. An important algorithm was the “U-Net”, which was modified from the fully convolutional network (FCN), which became the cornerstone of many newly developed models [35].

Segmentation or contouring operations are mainly influenced by the object’s edge information. Despite the successful extraction of edge features, U-Net still has its drawbacks. The most important of them is that it is unable to adequately predict high-level features for high-resolution edge information in the input image. U-Net uses skip connections to transmit high-resolution information. However, unlike the low-resolution features, the high-resolution edge information is transmitted to the skip connection after the pooling layer without passing through a convolution layer. Therefore, the U-Net tries to disproportionately extract high-resolution features from low-resolution information.

To overcome the limitations of the U-Net, this paper proposes AIM-Unet by redesigning it by placing convolutional layers of different filter sizes on the skip connection. AIM-Unet prevents duplication of low-resolution features from the residual path and extracts higher-resolution features with high-resolution edge information for large images. Compared to U-Net, AIM-Unet reveals and processes edge information and morphology features to a greater extent.

## 3. Materials and Methods

**Figure 1:**
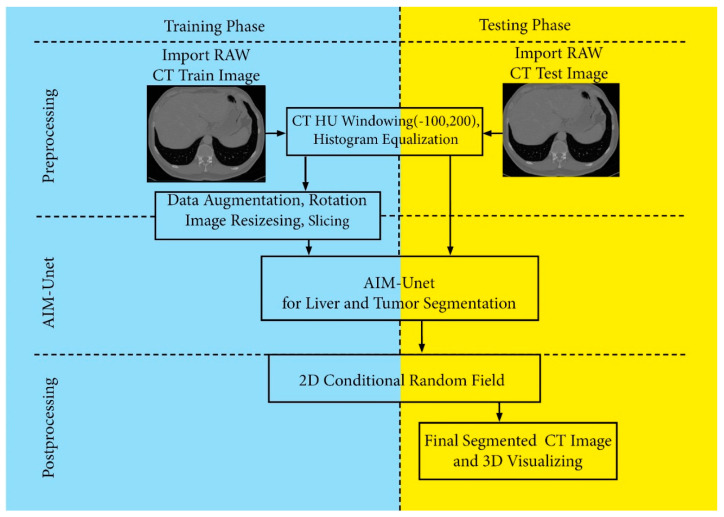
The schematic representation of the AIM-Unet model.

In this study, an Unet and Inception-based model is proposed for automatic segmentation of liver and tumor on abdominal CT images. The block diagram is showing the methodology of the proposed model, and is summarized in three phases: data preparation, training and testing, and segmentation (Figure 1).

### 3.1. Deep Learning Model Development

**Figure 2:**
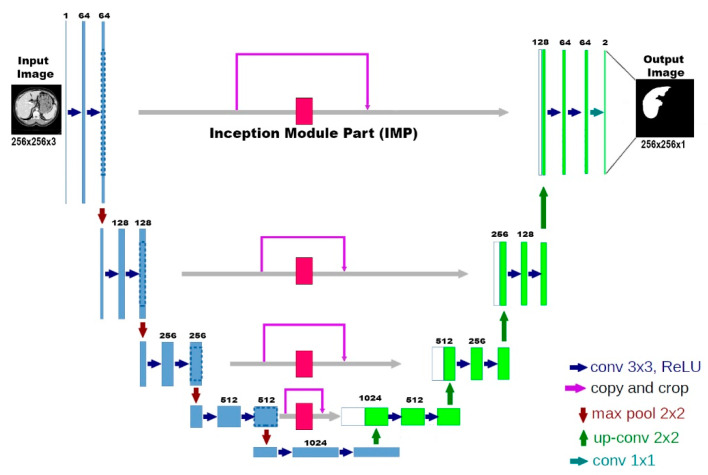
AIM-Unet architecture.

In the last few years, deep CNNs have achieved significantly good outcomes in medical image classification. The simplest way to improve the performance of deep CNNs is to increase their depth and width [36]. Szegedy et al. [37] proposed a deep CNN architecture called Inception that increases the width of each stage. There are multiple versions of Inception-Net. The versions of Inception-v2 [38], Inception-v3 [39,40], and Inception-v4 [41] are widely used in classification tasks. He et al. [42] Redundant connections introduced by MD have made it easy to train very deep networks. Combining both ideas, Inception-ResNet outperformed previous networks in applications. However, U-Net is one of the most preferred models in medical image segmentation. The model got its name from its resemblance to the letter “U”, as can be seen from its shape. The reason it is preferred is that it gives successful results, and the training period is short. The original U-Net model is a successful convolution network developed for biomedical image segmentation. U-Net basically consists of two parts: the encoder/downsizing path and the decoder/expansion path. Different versions of U-Net have also been developed by researchers. A few of these versions are Recurent Residual-Unet [43], Attention-Unet [44], and Attention Residual-Unet [45]. The model we propose for liver segmentation, AIM-Unet, is shown in Figure 2. This model consists of a combination of the U-Net model and a module of the Inception model. On all four levels of the skip connection, the output of the convolutional layer in the encoder part is concatenated with the output of the Inception module section and then transferred to the decoder. These feature maps are then concatenated with the output of the upsampling operation. The last convolution layer of the decoder part uses a Sigmoid activation function and a 1 × 1 convolution operation. In the output layer, if we were to perform multiple classifications, we would have to use the softmax function for the activation function. The output of the model is a 256 × 256 binary image. There are a total of 188 layers in our model, and the total number of parameters is 41,695,169. 41,672,129 of these parameters are trainable, and 23,040 are non-trainable. In the proposed model, the input, output dimensions, and filter numbers in the layers are given in Table 1.

### 3.2. Inception Module Part (IMP)

**Figure 3:**
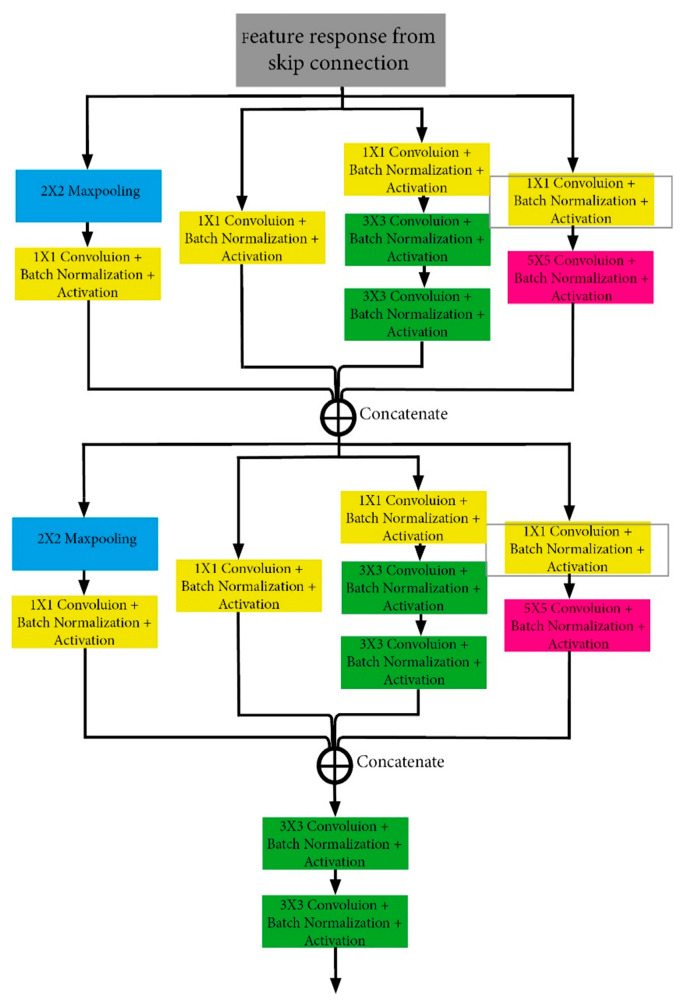
Diagram of inception module part (IMP).

In this study, the presented model for the liver segmentation was added to the Unet model, the module of the inception model, which includes the convolution and pooling layers, as shown in Figure 3. This module has been added to the skip link. The module we added processes the input data in 4 different branches. While only 1 × 1 convolution operation is performed in one arm, a 1 × 1 convolution operation is performed first, and then a 5 × 5 convolution in the second arm. In the third arm, first a 1 × 1 convolution operation is performed, and then a 3 × 3 convolution operation is performed twice. In the last arm, first the maximum pooling operation is performed, and then the 1 × 1 convolution operation is performed. After this four-branch operation is performed, the merge operation is applied to the outputs. These operations are repeated once again. It is then subjected to two 3 × 3 convolution and activation processes. We used the batch normalization and ReLu functions after each convolution operation. These processes are shown in detail in Figure 3.

## 4. Experiments

We have performed all the work on the datasets on a computer with the following configuration.

- CPU: Intel Core i7-10875H 2.3 GHZ
- GPU: 8 GB NVIDIA GeForce RTX 2080 Super with Max-Q Design.
- Memory: 16,384 Mb RAM

We used the Tensorflow 2.2.0 framework, which allows for dynamic stack size, to do the research. We also developed our application on the Spider 5.0 IDE, with Python 3.6 interpreter and Keras-nightly version 2.5.0. We chose the binary cross entropy function as the loss function for the measurement of accuracy and loss. We also observed IoU (Intersection Over Union), Dice loss, recall, and precision metrics during the training. The network architecture is based on the U-Net architecture and the Inception architecture. However, convolution and stack normalization layers are included in the skip connections of this study, and the number of layers has changed in the skip connections. Therefore, the network is trained from scratch each time. During the training, many experiments were carried out by adjusting the hyperparameters used in the network. The learning rate, batch size, epoch and dropout value, optimizer, loss function, and activation function were checked for different values and assignments. After much trial and error, a batch size of 3, epochs of 100, a validation division of 0.20 (after test data separation), and a learning rate of 0^−3^ were used. We saw that the Adaptive Moment Estimation (Adam) optimizer gave the best results. When we used early stopping for the validation loss (for patience = 5) in our trainings, we saw that the 95th epoch training was complete. Most values did not change after this epoch, and that is why we didn’t use early stops in the next training. We finished the training in the 100th epoch

### 4.1. Datasets

We use three liver image datasets to evaluate our model, two of which are the publicly available LiST dataset and the CHAOS dataset, and the other is the dataset we created. Most of the liver-free sections of patients in these datasets were discarded. Any patient’s data in the datasets is 512 × 512 × N in size. Here, the N number is called the number of slices and may vary according to the patient’s liver size. Each slice (abdominal) image of the patients was recorded as an image. 10% of patients were randomly assigned for testing across all datasets. Then, in the remaining patients, 30% were randomly assigned as validation data and 70% as test data. Each dataset was trained and tested separately. Details of the number of images used in datasets are given in Table 2.

#### 4.1.1. Patients and Data Acquisition Protocol

This study was carried out in accordance with the criteria determined by the Ministry of Health by obtaining the necessary permission from the Faculty of Medicine of Trakya University, dated 12 February 2020, and numbered 600-E.411005. The study was performed in accordance with the Helsinki Declaration and approved by the local ethics committee, numbered TÜTF-BAEK 2020-7254. In addition, patient data were collected and processed in accordance with Republic of Turkey Law No. 6698 on the Protection of Personal Data. In this study, data from 50 different patients (28 males and 22 females) aged between 25 and 83, some of whom were diseased and some of whom were healthy, were used. The dataset was created with the abdominal images of each patient, consisting of an average of 73 sections.

#### 4.1.2. Creating the Dataset (Our Dataset)

It is very difficult to obtain the amount of data that needs to be collected to solve a segmentation problem. By difficult, we mean time, hardware, and perhaps most importantly, the labeling of data. An ordinary eye will not be sufficient for tagging, localization, and positioning information of each pixel, especially in areas that require sensitive approaches such as biomedical or defense. For this, expert eyes and experience are needed in many related images.

Appropriate PET-CT images were stored from the hospital database in Digital Imaging and Processing in Medicine (DICOM) format. All stored images were resized to a resolution of 4.7 × 6.3 × 30 mm. The sections were examined one by one by the specialist medical doctor, and the anatomical liver tissue was marked. Liver areas (ROI) determined by expert radiologists were labeled 1, and non-hepatic areas were labeled 0. After the tagging process, the original images were added to the dataset as 512 × 512 × 3 pixels with a jpg extension with a 3-channel color depth, and the tagged images as a png extension with a single-color channel with 512 × 512 × 1 dimensions.

#### 4.1.3. The LiTS Image Dataset

The images in this dataset were collected from clinics and university hospitals in various countries around the world. IRCAD Institute Strasbourg, Hebrew University of Jerusalem, Tel Aviv University, Sheba Medical Center, Ludwig Maximilian University of Munich, Polytechnique and CHUM Research Center Montreal, and Radboud University Medical Center of Nijmegen are some of them. Most of the patients in this dataset suffer from liver diseases. The images of the patients include primary (i.e., originating in the liver-like hepatocellular carcinoma, HCC) and secondary (i.e., spreading to the liver-like colorectal cancer) tumor diseases, as well as metastases originating from breast and lung cancers. Images were acquired from different devices under different acquisition protocols. Therefore, the data has different resolutions, image quality, and contrast densities. The LiTS dataset contains 131 and 70 contrast-enhanced 3D abdominal CT scans for training and testing, respectively [46]. The number of sections in the images of these patients ranged from 42 to 1026, and the number of sections containing the liver ranged from 30 to 253. Each patient has an average of 150 images. The sections have different thicknesses between 0.45 mm and 6.0 mm. Data were downloaded in nfii file format, and images without liver were extracted for a faster conclusion of the model. In the training and test sets of the liver, volumes show a normal distribution and are similar to known distributions. There are 19,433 images in total in the dataset, and the usage distribution of these images is shown in detail in Table 2.

#### 4.1.4. CHAOS Dataset

The images in this dataset were collected by the Department of Radiology at Dokuz Eylül University. The dataset includes original CT images of 40 patients and images marked by expert radiologists. Of these patients, 22 were men and 18 were women [47]. In addition, the age range of the patients ranged from 18 to 63 years. Patient images do not contain any pathological abnormalities (tumors, metastases). So the images contain only healthy abdominal organs. There are 2975 images in total in the dataset, and the distribution of use of these images is shown in detail in Table 2. The number of slices for each patient varies between 77 and 105, and an average patient has 90 images. Images are in a DICOM format with 512 × 512 resolution. The slice thickness of each image ranges from 3.0 to 3.2 mm. As in the LiST dataset, the liver sizes in this dataset show differences. The reason for this is that the anatomical structure of each patient is different.

#### 4.1.5. 3DIRCADb Dataset

This dataset is a subset of patients 28-47 from the LiST dataset, which consists of 131 patient records. A total of 20 patients are included in the 3DIRCADb dataset. 16 of these patients have tumor images. The total number of slices with tumors is 588.

### 4.2. Data Preparation and Processing

**Figure 4:**
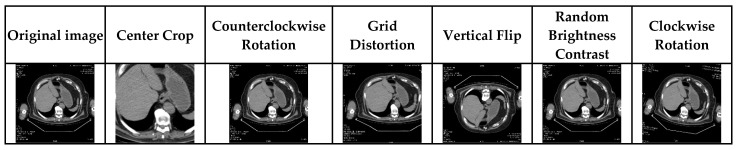
Outputs of images obtained by data augmentation method.

Pre-processing was undertaken in a slice-wise fashion. In the first step, liver-free slices of each patient in the dataset were excluded from the dataset. To exclude irrelevant organs and objects, Hounsfield unit values were automatically windowed in the range [−100, 200] with the help of a function, and then we increased the contrast through histogram equalization. The data was converted to jpg and png formats to make it ready for preprocessing, resampling, and post-structuring training. When training a model, data augmentation is important to effectively train the network if the dataset is small. For this reason, data augmentation is applied by applying different affine deformations to the existing training images [48]. Especially when creating medical image datasets, the labeling process of the data is very laborious and takes a long time, so the number of images in the dataset is insufficient. The small number of training pairs raises the problem of overfitting. We applied center crop, clockwise rotation, counterclockwise rotation, grid distortion, vertical flip, and random brightness contrast affine to each image in our dataset. We created an extra 6 different views from each image. In this way, the data set has been increased 7 times. Figure 4 contains images of the results of affine applied to an image. The original dimensions of the images in the dataset are 512 × 512. Since using images at these sizes will tire the GPU and to avoid memory errors, the images have been reduced to 256 × 256. The images were also normalized between 0 and 1.

### 4.3. Post-Processing

While any image is predicted or tested by the model, each pixel of the image is independently classified as liver or background. The image value obtained as a result of the estimation is sent to a generated threshold function. The function examines the value of each pixel individually. If the pixel value is greater than 0.5, that pixel is considered a liver and assigns 1 to that pixel’s value. If the pixel value is less than 0.5, it considers this pixel as the background and assigns the value 0. This process is similarly applied to the tumor image.

If a 3-dimensional view is to be obtained from all images of the patient, after the above steps are applied to all slices of the patient, these slices are combined and mesh (Laplacian smoot, Faces orientation) operations are applied. The 3D image of a patient we obtained is in last figure.

### 4.4. Metrics

**Figure 5:**
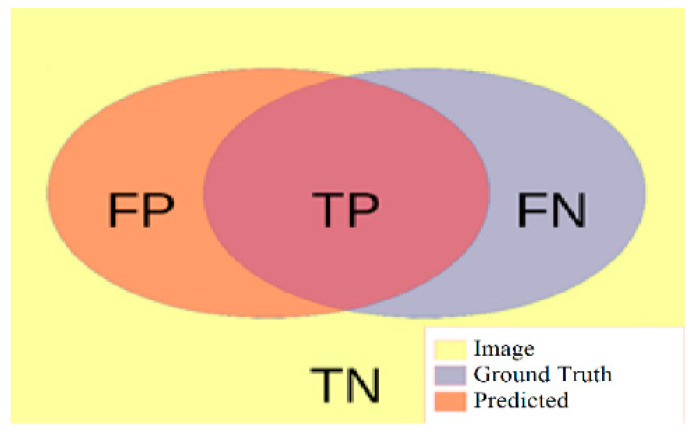
A graphical illustration of True Positive (TP) represent the pixels considered as being in the pre-dicted liver and being really in the ground truth liver, False Positive (FP) are the pixels considered by the segmentation in the liver, but which in reality are not part of it, True Negative (TN) are the pixels outside the liver both in the segmentation and the ground truth, and False Negative (FN) are the pixels of the liver that the segmentation has classified when comparing predicted results.

Various measurement performances are taken into account to analyze the experimental results [49,50]. Accuracy (AC), recall (sensitivity) (RE), prevalence (PV), specificity (SP), precision (PR), Dice similarity coefficient (DSC), and Jaccard similarity coefficient (JSC) measurement performances are some of them. To calculate these performance measures, a confusion matrix is used, which includes the variables True Positive (TP), False Positive (FP), True Negative (TN), and False Negative (FN) in Figure 5.

The overall accuracy value was calculated using Equation (1), and recall is calculated using Equation (2).

(1) $$AC=\frac{TP+TN}{TP+TN+FP+FN}\;\;$$

(2) $$RE=\frac{TP}{TP+FN}\;$$

Furthermore, the precision [51] value is calculated using the following Equation (3).

(3) $$PR=\frac{TP}{TP+FP}\;\;\;\;\;\;\;$$

Cross-entropy loss is the loss function that is widely employed in semantic segmentation tasks. The cross-entropy loss examines each pixel one by one and compares the predicted results (probability distribution vector) for each pixel category with the heat-coded label vector. When there are only two categories, a binary cross entropy loss, called BCE loss, is used and represented as Equation (4). We chose the binary croossentropy function as the loss function when training the datasets. Cross-entropy [4] can be defined as a measure of the difference between two probability distributions for a given random variable or set of events. It is commonly used to classify the objects and the segmentation levels, if pixel works properly.

Binary cross-entropy is defined as:

(4) $$L_{BCE}=-\left[P\log\left(G\right)+\left(1-P\right)\log\left(1-G\right)\right]$$

Here, P is the predicted image by the prediction, and G is the ground truth image.

We can consider Dice score as the matching level between predicted and ground truth image. The range of a Dice score is a value between 0 and 1. The higher the score, the better the performance. If this value is zero, it indicates that there is no relationship between the predicted image and the ground truth image. It is clear that the maximum of Dice score is one, if the predicted image and the ground truth image are completely overlapping. In addition to the calculation of binary precision, the threshold parameter, which we set to 0.5, is calculated. Each predicted pixel is compared with the threshold. If the pixel value is less than or equal to the threshold value, 0 is assigned to the value of that pixel, if the pixel value is greater than the threshold value, that pixel value is assigned 1.

The Dice coefficient is calculated using Equation (5), and Dice loss is calculated using Equation (6).

(5) $$DSC=\frac{2\left|P\cap G\right|}{\left|P\right|+\left|G\right|}\;\;$$

(6) $$DL=1-\frac{2\left|P\cap G\right|}{\left|P\right|+\left|G\right|}$$

where P is the image predicted by our proposed model, and G is the ground truth image.

Jaccard index is very popular and used as a similarity index for binary data, as shown in the following form.

(7) $$JSC=\frac{P\cap G}{P\cup G}$$

We also calculated the mean IoU, recall, and precision metrics as well as the Dice score metric. These metric values are given in Table 3 and Table 4 for 3 datasets.

**Figure 6:**
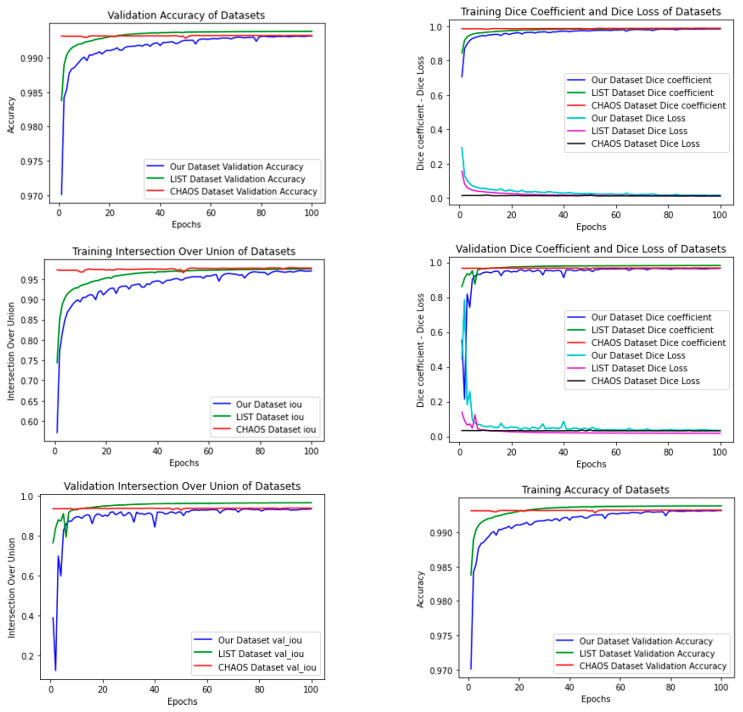
The figures above show the changes in Dice score, accuracy, and IoU values during each epoch of the AIM-Unet model that we trained on three different datasets.

**Figure 7:**
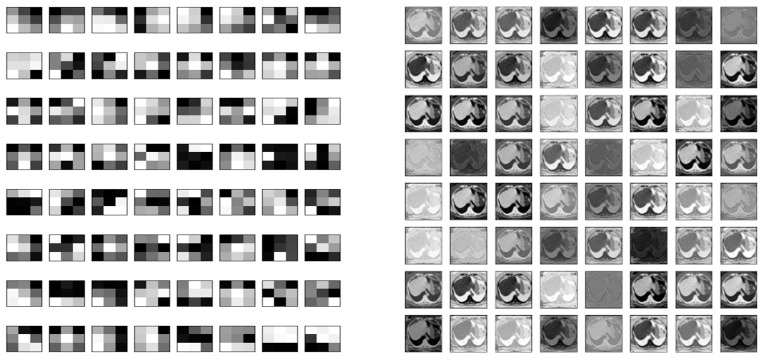
The figures above show 64, 3 × 3 filters in the first convolution layer and 64, 256 × 256 filters in the fourth layer during training.

The aim of this study is to reveal the ability of different sized filters in the Inception module added to the U-Net model to automatically extract the liver region. The evaluation of the method was measured by comparing the area estimated by a radiologist with the actual area. The most commonly used measurement values are Dice score and Jaccard index (IOU). The changes to these values in our model are as shown in Figure 6. Figure 6 shows the decimation of the validation errors after the Adam optimizer, as we used the cause of the ted concussions and the refresh of the learning rate. Some masks that we obtained as a result of training the model are shown in Figure 7. To measure the success of our proposed model, we trained the network only with our dataset and then tested it separately on the LiST and CHAOS datasets. Next, we repeat this procedure for the other dataset as well, and we report the results on Table 3. The values in Table 3 show the test results of the proposed model after training on each dataset separately. The numbers of images used in the training and test data sets are given in Table 2. If we pay attention to Table 4, the results trained on the LiST dataset and tested on our dataset have the largest standard deviations. The reason for this is that the image in our data set differs as a result of morphological operations. As seen from Table 4, the most successful test results are from the model trained on the CHAOS dataset. However, the test results of the proposed model trained on our dataset and tested on the Chaos dataset are very close to the highest values. Also, looking at the data in the table, it is seen that the AIM-Unet model has achieved successful results in all datasets.

As can be seen from the test set results in Table 4, the model we propose has very good results on three datasets. The test results in Table 4 obtained from the three data sets show that the model can be easily used in daily routine clinical applications.

**Figure 8:**
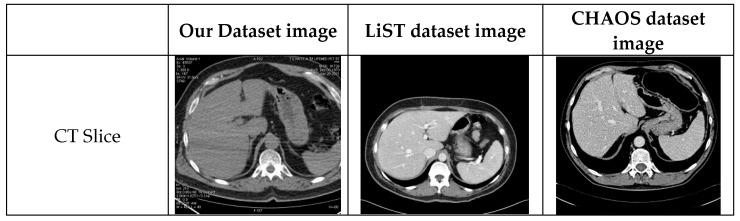

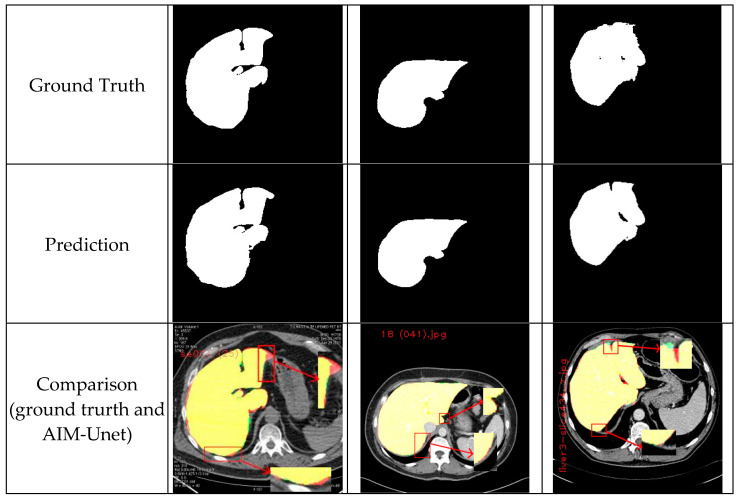
Performance of the proposed model (AIM-Unet) on images of datasets. The red areas in the comparison image show the regions that our model missed, the green areas are the areas that the model predicted incorrectly, and the yellow areas are the regions that the model predicted correctly.

**Figure 9:**
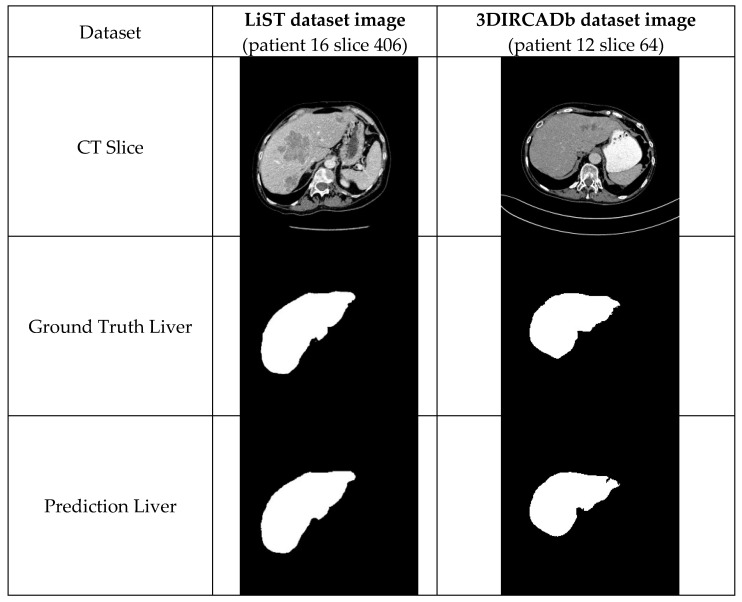

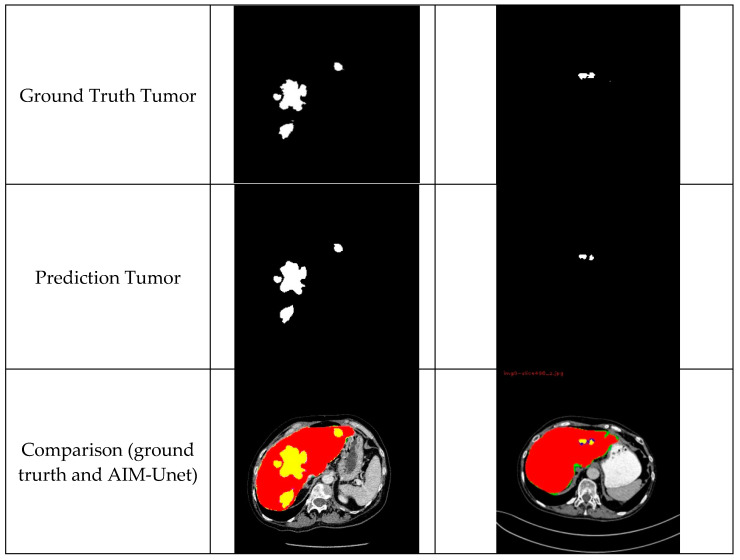
Performance of the proposed model (AIM-Unet) on images of datasets. The green areas in the comparison image show the regions that our model missed for the liver area, the blue areas are the regions that the model missed for the tumor area. The red areas show the region where our model correctly predicted the liver, and the yellow areas show the tumor areas that our model predicted correctly.

In this part of the study, we discuss and analyze the computed tomography results in the datasets to evaluate the performance of the proposed model. The results estimated by AIM-Unet from images on different datasets are shown in Figure 8. The red areas in the comparison image show the regions that our model missed, the green areas are the areas that the model predicted incorrectly, and the yellow areas are the regions that the model predicted correctly. The estimation results of liver and tumor regions in the LiST and 3DIRCADb datasets by AIM-Unet are shown in Figure 9.

The average Dice score and Jaccard index values of the proposed model for liver segmentation are given in Table 5, and those for tumor segmentation in Table 6. As can be seen from Table 5, the model we propose has higher values than other models in terms of Dice score and Jaccard index. This shows that the use of different filters in the model produces more successful features and makes the model more successful. However, the model we proposed on the CHAOS dataset and the dataset we created, has been the most successful model. In addition, when we look at the Jaccard indexes, it was the model we recommended that showed the most successful performance on 3 datasets. While the model proposed for tumor segmentation in Table 6 achieved very successful results on the LiST dataset, its success remained low in the 3DIRCADb dataset. The reason for this is that there are more tumor images in the LIST dataset, and the model learns better from this extra image.

The number of patients required to train a segmentation model depends on the variability in the data, the completeness of the data with respect to missing labels, and the AI model used. A model for tumor segmentation usually requires more patient data than OARs because tumor shape and location are more variable than in normal anatomy. Currently, state-of-the-art CNN-based shaping models typically consist of more than 100 patients. However, models of 50–100 patients have also been shown to segment OARs with reasonable accuracy. Information on the patient data sets used in our study is given in Table 2. The variability in the training data should reflect the variability of the clinical data for which the model will be used. For example, if the model will be used for different imaging acquisition protocols or devices, the training set should contain all of these types of data [62]. Finishing operations such as bonded component selection, hole filling, or smoothing can be performed to obtain more clinically relevant contours. Validation and test sets typically contain about 20 patients. A minimum of 10 patients is recommended but should be increased if there are large differences in outcomes. The test sets used in our study are given in Table 2.

Both IoU and DSC are used to calculate the overlap of baseline truth masks and U-Net generated images, indicating the degree of similarity. High DSC and IoU values correspond to correct segmentation (Table 5).

Contour definition accuracy in radiotherapy is also evaluated using DSC. Among CNN architectures adapted for medical imaging, U-Net and its variations are the most popular among researchers, and techniques have been devised to deal with smaller datasets, a practical limitation in many medical imaging applications [63].

## 5. Conclusions

**Figure 10:**
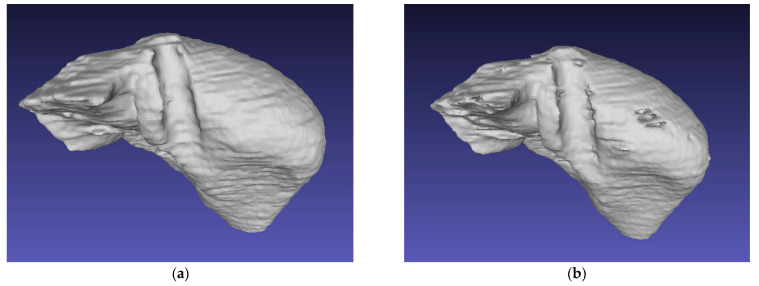
3D visual liver labeled by specialist radiologists (**a**) and segmentation results with a proposed model (**b**) (patient 16 in the LiST dataset).

In this study, we examined how the Inception model’s different filtering module will be added to the skip connection of the U-Net model and how it will affect the segmentation by taking advantage of the local features in the CT abdomen images. We applied this study to three different datasets. As it can be understood from the results, we saw that the different filter module we added helped us learn more local features than the standard network. Our model is an end-to-end trainable model. It does not need pre-trained weights. We found that our model is more complex than the standard U-Net model. The entire algorithm was able to segment a slice in 1.12 s using a typical desktop computer. The average time required for a CT cross-sectional image to be segmented by a specialist is 2 min. Therefore, our proposed method is approximately 100 times faster than manual segmentation at acceptable accuracy levels. As a result of our experiments on three data sets, the Dice values we obtained for liver segmentation show that it is 97.38 for our dataset, 95.77 for LiST, and 97.86 for CHAOS. These quantitative metrics demonstrate that our method can accurately segment the liver from 2D abdominal CT images. Figure 10 shows the 3D rendering of the 2D abdominal CT images of patient 16 in the LiST dataset, which were estimated from the proposed model. Moreover, the results show that this proposed model provides better performance not only during training but also during testing. However, the different-sized convolution layers we add to the skip connections increase the number of parameters of the model too much. This increases the training time of the model and causes the GPU to become overtired. Since the estimation time was not specified in previous studies, it is not possible to make a definite judgment about the estimation time of our model. In the future, we want to apply the same architecture to liver tumors, to 3D render the model, and apply it to different datasets.

Artificial intelligence is a growing class of tools with the potential to impact healthcare. The delineation of organs at risk (OAR) and target volumes, one of the starting points of radiotherapy treatment planning, is very important for accurate treatment planning. The current practice of treatment planning is largely a manual process that is time-consuming and labor-intensive, usually requiring hours or days for a case. Therefore, the quality of planning depends heavily on the planner’s contouring experience. To increase speed and consistency, automatic segmentation has become a major research focus. The use of artificial intelligence, including deep learning to automate radiotherapy planning, is expected to increase in the future. Clinicians must verify the data obtained to ensure the accuracy of patient treatment [64].

The AIM-Unet has very good potential for further development. In addition, a comparison of the data obtained with AIM-Unet in radiotherapy and manual contouring data is considered.

## Figures and Tables

**Table 1:** The input, output and number of filters for proposed model (AIM-Unet).

Encoder Part					Inception Module Part					Decoder Part				
Block	Input	Layer (Filter Size)	Output	Filters	Block	Input	Layer	Output	Filters	Block	Input	Layer (Filter Size)	Output	Filters
Encoder 1	(256,256)	Conv2D (3,3)	(128,128)	64	Skip Connection 1	(256,256)	IMP 1	(256,256)	128	Decoder 1	(128,128)	Conv2D (3,3)	(256,256)	64
		Conv2D (3,3)										Conv2D (3,3)		
Encoder 2	(128,128)	Conv2D (3,3)	(64,64)	128	Skip Connection 2	(128,128)	IMP 2	(128,128)	256	Decoder 2	(64,64)	Conv2D (3,3)	(128,128)	128
		Conv2D (3,3)										Conv2D (3,3)		
Encoder 3	(64,64)	Conv2D (3,3)	(32,32)	256	Skip Connection 3	(64,64)	IMP 3	(64,64)	512	Decoder 3	(32,32)	Conv2D (3,3)	(64,64)	256
		Conv2D (3,3)										Conv2D (3,3)		
Encoder 4	(32,32)	Conv2D(3,3)	(16,16)	512	Skip Connection 4	(32,32)	IMP4	(32,32)	1024	Decoder 4	(16,16)	Conv2D (3,3)	(32,32)	512
		Conv2D (3,3)										Conv2D (3,3)		
Base	(16,16)	Conv2D (3,3)	(16,16)	1024						Base	(16,16)	Conv2D (3,3)	(16,16)	1024

**Table 2:** Distribution of images in datasets.

Dataset	Training Slices	Validation Slices	Test Slices	Total
CHAOS	1935	828	212	2975
LiST	13171	5644	618	19433
Our Dataset	2362	1011	254	3627

**Table 3:** Measurement performances of AIM-Unet on test datasets.

Dataset	Accuracy (%)	Recall (%)	Precision (%)	Mean IoU (%)
Our Dataset	99.54 ± 0.27	96.35 ± 2.64	98.47 ± 1.23	97.21 ± 1.46
CHAOS	99.75 ± 0.10	96.39 ± 6.23	99.69 ± 0.55	97.91 ± 3.13
LiST	99.48 ± 0.48	95.12 ± 6.15	96.78 ± 3.78	95.82 ± 3.52

**Table 4:** Test results of AIM-Unet model trained with different datasets.

Model Name	Train Dataset	Test Dataset					
		Our Dataset		LiST		CHAOS	
		Dice (%)	Jaccard (%)	Dice (%)	Jaccard (%)	Dice (%)	Jaccard (%)
AIM-Unet	Our Dataset	97.38 ± 1.63	94.95 ± 3.00	93.60 ± 7.49	88.60 ± 9.10	97.64 ± 6.74	95.91 ± 7.81
	LiST	77.43 ± 27.60	69.61 ± 28.96	95.77 ± 5.16	92.22 ± 6.90	94.12 ± 5.97	89.38 ± 8.70
	CHAOS	97.06 ± 3.89	94.53 ± 6.02	93.46 ± 9.46	88.64 ± 10.49	**97.86 ± 4.65**	**96.10 ± 6.21**

**Table 5:** The comparison of the proposed method (AIM-Unet) with other state-of-the-art architectures on datasets for liver segmentation.

Model	Dataset	Dice (%)	Jaccard (%)
Ronneberger et al. [25] *	LiST	95.2	90.8
Lin et al. *	LiST	90.9	86.7
Li et al. [46]	LiST	96.5	92.6 *
Yuan et al. [52]	LiST	96.7	92.9 *
Thi et al. [31]	LiST	96.0	90.4
Unet	LiST	87.86 ± 20.28	82.10 ± 21.05
AIM-Unet	LiST	95.77 ± 5.16	92.22 ± 6.90
Ronneberger et al. [25] *	CHAOS	74.75	
Thi et al. [31]	CHAOS	94.92	
Mourya et al. [53]	CHAOS	97.0 ± 0.03	
Unet	CHAOS	68.30 ± 33.96	60.35 ± 33.24
AIM-Unet	CHAOS	**97.86 ± 4.65**	**96.10 ± 6.21**
Unet	Our Dataset	46.64 ± 35.52	37.69 ± 31.72
AIM-Unet	Our Dataset	97.38 ± 1.63	94.95 ± 3.00

Note: * the result are obtained from [32].

**Table 6:** The comparison of the proposed method (AIM-Unet) with other state-of-the-art architectures on datasets for tumor segmentation.

Model	Dataset	Dice (%)
AIM-Unet	3DIRCADb	65.5 ± 12.9
Li et al. [54]	3DIRCADb	66.3
Omar et al. [55]	3DIRCADb	75.0
Christ et al. [56]	3DIRCADb	56.0
Chen et al. [57]	LiST	66.6
Vorontsov et al. [58]	LiST	66.1
Bi et al. [59]	LiST	64.5
Liu et al. [60]	LiST	63.4
Zhang et al. [61]	LiST	58.7 ± 28.3
Li et al. [54]	LiST	74.1
AIM-Unet	LiST	**75.6 ± 13.4**

## Data Availability

Our code and experimental data are publicly available at: https://github.com/frtozcan/trakyauniv. (accsessed on 4 January 2023).
